# (*E*)-3-Hydr­oxy-13-methyl-16-[4-(methyl­sulfan­yl)benzyl­idene]-7,8,9,11,12,13,15,16-octa­hydro-6*H*-cyclo­penta­[*a*]phen­an­­­­thren-17(14*H*)-one

**DOI:** 10.1107/S1600536808040877

**Published:** 2008-12-10

**Authors:** B. Gunasekaran, R. Murugan, S. Sriman Narayanan, V. Manivannan

**Affiliations:** a Department of Physics, AMET University, Kanathur, Chennai 603 112, India; b Department of Analytical Chemistry, University of Madras, Guindy Campus, Chennai 600 025, India; c Reader in Physics, Presidency College, Chennai 600 005, India

## Abstract

In the title compound, C_26_H_28_O_2_S, the dihedral angles between the mean plane of the five membered ring and the 4-(methyl­sulfan­yl)benzyl­idine ring in the two crystallographically independent mol­ecules are 34.05 (10) and 40.53 (15)°. The packing is stabilized by inter­molecular O—H⋯O and C—H⋯O inter­actions.

## Related literature

For the biological activity of testosterone derivatives, see: Alvarez-Ginarte *et al.* (2005 ▶). For puckering parameters, see: Cremer & Pople (1975 ▶). For related structures, see: Suitchmezian *et al.* (2007 ▶); Ye (2007 ▶).
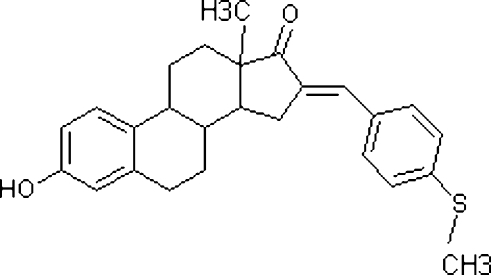


## Experimental

### 

#### Crystal data

C_26_H_28_O_2_S
*M* *_r_* = 404.54
Orthorhombic, 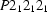

*a* = 11.9654 (3) Å
*b* = 13.0262 (4) Å
*c* = 27.9441 (8) Å
*V* = 4355.5 (2) Å^3^
*Z* = 8
Mo *K*α radiation
μ = 0.17 mm^−1^
*T* = 293 (2) K
0.25 × 0.20 × 0.15 mm

#### Data collection

Bruker Kappa APEXII diffractometer
Absorption correction: multi-scan (*SADABS*; Sheldrick, 1996 ▶) *T*_min_ = 0.959, *T*_max_ = 0.975
21727 measured reflections
6847 independent reflections
4775 reflections with *I* > 2σ(*I*)
*R*_int_ = 0.040
θ_max_ = 24.1°

#### Refinement

*R*[*F* ^2^ > 2σ(*F* ^2^)] = 0.049
*wR*(*F* ^2^) = 0.142
*S* = 1.06
6847 reflections
529 parameters
H-atom parameters constrained
Δρ_max_ = 0.38 e Å^−3^
Δρ_min_ = −0.30 e Å^−3^
Absolute structure: Flack (1983 ▶), 2995 Friedel pairs
Flack parameter: −0.06 (11)

### 

Data collection: *APEX2* (Bruker, 2004 ▶); cell refinement: *SAINT* (Bruker, 2004 ▶); data reduction: *SAINT*; program(s) used to solve structure: *SHELXS97* (Sheldrick, 2008 ▶); program(s) used to refine structure: *SHELXL97* (Sheldrick, 2008 ▶); molecular graphics: *PLATON* (Spek, 2003 ▶); software used to prepare material for publication: *SHELXL97*.

## Supplementary Material

Crystal structure: contains datablocks global, I. DOI: 10.1107/S1600536808040877/bt2822sup1.cif

Structure factors: contains datablocks I. DOI: 10.1107/S1600536808040877/bt2822Isup2.hkl

Additional supplementary materials:  crystallographic information; 3D view; checkCIF report

## Figures and Tables

**Table 1:** Hydrogen-bond geometry (Å, °)

*D*—H⋯*A*	*D*—H	H⋯*A*	*D*⋯*A*	*D*—H⋯*A*
O1—H1⋯O2^i^	0.82	1.95	2.746 (3)	163
C10—H10⋯O2^i^	0.93	2.53	3.179 (4)	127
O3—H3*A*⋯O4^ii^	0.82	2.03	2.830 (4)	166
C36—H36⋯O4^ii^	0.93	2.47	3.209 (4)	136

Symmetry codes: (i) 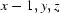; (ii) 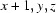.

**Fig. 1.**
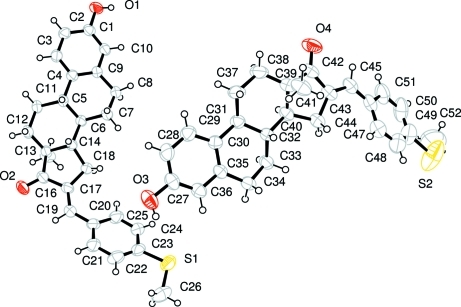
The molecular structure of the title compound, with atom labels and 50% probability displacement ellipsoids for non-H atoms.

**Fig. 2.**
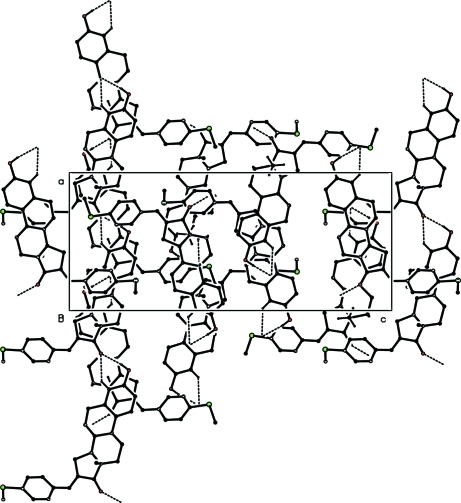
The packing of the title compound, viewed down the b axis. Hydrogen bonds are shown as dashed lines.

## supplementary crystallographic information

### Comment

Testosterone derivatives exhibit a high level of biological activity and have been widely used as hormone treatments (Alvarez-Ginarte *et al.*, 2005).

The five membered rings C13/C14/C16/C17/C18, C39/C40/C42/C43/C44 and 4-(methylsulfanyl)benzylidine rings of the two independent molecules are planar. The C11—C14/C6/C5 and C4—C9 in molecule(I), C37—C40/C32/C31 and C30—C35 in molecule(II) rings have chair conformation (Cremer & Pople, 1975). The dihedral angle between C8/C7/C4/C5 and C11/C12/C14/C6 planes in molecule (I), C33/C34/C30/C31 and C32/C40/C37/C38 planes in molecule (II) are 4.62 (13) and 4.23 (14) °. The molecular structure is stabilized by weak intramolecular C—H···O interactions and the crystal packing   through intermolecular O—H···O and C—H···O interactions.

### Experimental

1.0 mol of 4-(methylsulfanyl) benzaldehyde (1.0 g) was dissolved in methanol containing 0.1 g of sodium hydroxide. To this solution 1.0 mol of estrone (0.56 g) was added. The solution was  stirred   overnight at room temperature. The crude solid was filtered off and then recrystallized in ethanol.

### Refinement

H atoms were positioned geometrically and refined using a riding model with O—H = 0.82Å and *U*_iso_(H) = 1.2Ueq(O), C—H = 0.93Å and *U*_iso_(H) = 1.2Ueq(C) for aromatic C—H, C—H = 0.97Å and *U*_iso_(H) = 1.2Ueq(C) for CH_2_, C—H = 0.96Å and *U*_iso_(H) = 1.5Ueq(C) for CH_3_.

### Crystal data

**Table:**

C_26_H_28_O_2_S	*F*(000) = 1728
*M**_r_* = 404.54	*D*_x_ = 1.234 Mg m^−^^3^
Orthorhombic, *P*2_1_2_1_2_1_	Mo *K*α radiation, λ = 0.71073 Å
Hall symbol: P 2ac 2ab	Cell parameters from 4404 reflections
*a* = 11.9654 (3) Å	θ = 1.5–24.1°
*b* = 13.0262 (4) Å	µ = 0.17 mm^−^^1^
*c* = 27.9441 (8) Å	*T* = 293 K
*V* = 4355.5 (2) Å^3^	Block, pale yellow
*Z* = 8	0.25 × 0.20 × 0.15 mm

### Data collection

**Table:**

Bruker Kappa APEXII diffractometer	6847 independent reflections
Radiation source: fine-focus sealed tube	4775 reflections with *I* > 2σ(*I*)
graphite	*R*_int_ = 0.040
Detector resolution: 0 pixels mm^-1^	θ_max_ = 24.1°, θ_min_ = 1.5°
ω and φ scans	*h* = −13→13
Absorption correction: multi-scan (*SADABS*; Sheldrick, 1996)	*k* = −14→14
*T*_min_ = 0.959, *T*_max_ = 0.975	*l* = −23→32
21727 measured reflections	

### Refinement

**Table:**

Refinement on *F*^2^	Secondary atom site location: difference Fourier map
Least-squares matrix: full	Hydrogen site location: inferred from neighbouring sites
*R*[*F*^2^ > 2σ(*F*^2^)] = 0.049	H-atom parameters constrained
*wR*(*F*^2^) = 0.142	*w* = 1/[σ^2^(*F*_o_^2^) + (0.0773*P*)^2^] where *P* = (*F*_o_^2^ + 2*F*_c_^2^)/3
*S* = 1.06	(Δ/σ)_max_ < 0.001
6847 reflections	Δρ_max_ = 0.38 e Å^−^^3^
529 parameters	Δρ_min_ = −0.29 e Å^−^^3^
0 restraints	Absolute structure: Flack (1983), 2995 Friedel pairs
Primary atom site location: structure-invariant direct methods	Flack parameter: −0.06 (11)

### Fractional atomic coordinates and isotropic or equivalent isotropic displacement parameters (Å^2^)

**Table:**

	*x*	*y*	*z*	*U*_iso_*/*U*_eq_	
S2	−0.2835 (2)	0.45026 (18)	−0.20650 (6)	0.1751 (10)	
C52	−0.3659 (6)	0.3576 (7)	−0.2034 (3)	0.197 (4)	
H52A	−0.3443	0.3135	−0.1775	0.296*	
H52B	−0.3639	0.3198	−0.2329	0.296*	
H52C	−0.4403	0.3825	−0.1980	0.296*	
S1	0.68074 (10)	0.04257 (9)	0.06634 (4)	0.0772 (4)	
C10	0.0077 (3)	−0.1662 (3)	0.39329 (13)	0.0480 (9)	
H10	−0.0452	−0.1752	0.3693	0.058*	
C1	−0.0260 (2)	−0.1691 (3)	0.44008 (13)	0.0457 (9)	
C4	0.1992 (3)	−0.1388 (3)	0.41632 (11)	0.0419 (8)	
O1	−0.13439 (17)	−0.1845 (2)	0.45361 (9)	0.0603 (7)	
H1	−0.1753	−0.1795	0.4302	0.090*	
C11	0.4053 (2)	−0.1657 (3)	0.43880 (12)	0.0490 (9)	
H11A	0.3955	−0.2396	0.4396	0.059*	
H11B	0.3901	−0.1394	0.4706	0.059*	
C18	0.5165 (2)	−0.1240 (3)	0.29171 (11)	0.0522 (10)	
H18A	0.4995	−0.1895	0.2769	0.063*	
H18B	0.4896	−0.0690	0.2713	0.063*	
C13	0.5510 (2)	−0.1717 (3)	0.37447 (12)	0.0421 (8)	
C2	0.0528 (3)	−0.1572 (3)	0.47588 (12)	0.0487 (9)	
H2	0.0315	−0.1587	0.5079	0.058*	
C5	0.3214 (3)	−0.1193 (3)	0.40352 (11)	0.0422 (8)	
H5	0.3319	−0.0447	0.4052	0.051*	
C3	0.1637 (3)	−0.1429 (3)	0.46359 (11)	0.0464 (9)	
H3	0.2164	−0.1358	0.4878	0.056*	
C14	0.4667 (2)	−0.1169 (3)	0.34152 (11)	0.0405 (8)	
H14	0.4708	−0.0441	0.3502	0.049*	
C16	0.6593 (3)	−0.1357 (3)	0.35210 (12)	0.0499 (9)	
C8	0.1480 (3)	−0.1465 (4)	0.32801 (11)	0.0582 (11)	
H8A	0.1443	−0.2155	0.3150	0.070*	
H8B	0.0928	−0.1053	0.3114	0.070*	
O2	0.74945 (18)	−0.1295 (2)	0.37285 (9)	0.0679 (8)	
C22	0.7872 (3)	0.0394 (3)	0.15450 (14)	0.0642 (11)	
H22	0.8416	0.0826	0.1419	0.077*	
C20	0.7142 (3)	−0.0535 (3)	0.22240 (12)	0.0486 (9)	
C6	0.3473 (2)	−0.1495 (3)	0.35240 (11)	0.0410 (8)	
H6	0.3428	−0.2244	0.3499	0.049*	
C12	0.5265 (2)	−0.1411 (3)	0.42538 (12)	0.0523 (10)	
H12A	0.5397	−0.0681	0.4292	0.063*	
H12B	0.5766	−0.1774	0.4468	0.063*	
C19	0.7213 (3)	−0.0814 (3)	0.27246 (12)	0.0523 (9)	
H19	0.7919	−0.0765	0.2862	0.063*	
C24	0.6231 (3)	−0.0568 (4)	0.14629 (14)	0.0676 (12)	
H24	0.5646	−0.0807	0.1274	0.081*	
C17	0.6397 (3)	−0.1134 (3)	0.30144 (12)	0.0474 (9)	
C9	0.1186 (2)	−0.1502 (3)	0.38065 (12)	0.0433 (8)	
C7	0.2624 (3)	−0.1029 (3)	0.31823 (12)	0.0552 (10)	
H7A	0.2608	−0.0289	0.3222	0.066*	
H7B	0.2838	−0.1177	0.2855	0.066*	
C23	0.7015 (3)	0.0076 (3)	0.12637 (13)	0.0556 (10)	
C25	0.6286 (3)	−0.0864 (3)	0.19251 (14)	0.0644 (11)	
H25	0.5738	−0.1298	0.2046	0.077*	
C15	0.5518 (3)	−0.2884 (3)	0.36806 (14)	0.0617 (11)	
H15A	0.5612	−0.3047	0.3348	0.093*	
H15B	0.4823	−0.3164	0.3792	0.093*	
H15C	0.6123	−0.3174	0.3861	0.093*	
C21	0.7946 (3)	0.0080 (3)	0.20193 (14)	0.0633 (11)	
H21	0.8553	0.0291	0.2203	0.076*	
C26	0.8118 (4)	0.0872 (4)	0.04740 (15)	0.0892 (15)	
H26A	0.8357	0.1421	0.0679	0.134*	
H26B	0.8063	0.1118	0.0151	0.134*	
H26C	0.8651	0.0322	0.0488	0.134*	
C36	0.4278 (3)	0.6531 (3)	0.12345 (13)	0.0516 (9)	
H36	0.4827	0.6492	0.0999	0.062*	
O3	0.5683 (2)	0.6698 (3)	0.18447 (10)	0.0758 (8)	
H3A	0.6091	0.6755	0.1610	0.114*	
C32	0.0900 (2)	0.6558 (3)	0.07867 (11)	0.0424 (8)	
H32	0.1021	0.7285	0.0714	0.051*	
C40	−0.0314 (2)	0.6301 (3)	0.06840 (12)	0.0454 (8)	
H40	−0.0426	0.5599	0.0800	0.055*	
C30	0.2331 (3)	0.6538 (3)	0.14494 (12)	0.0444 (8)	
C31	0.1107 (2)	0.6394 (3)	0.13184 (12)	0.0487 (9)	
H31	0.0940	0.5670	0.1380	0.058*	
C27	0.4588 (3)	0.6670 (3)	0.16988 (14)	0.0571 (10)	
O4	−0.3110 (2)	0.6600 (2)	0.09749 (10)	0.0798 (9)	
C37	0.0296 (3)	0.7010 (4)	0.16343 (15)	0.0713 (13)	
H37A	0.0462	0.7736	0.1604	0.086*	
H37B	0.0408	0.6818	0.1966	0.086*	
C44	−0.0779 (3)	0.6321 (3)	0.01750 (12)	0.0517 (9)	
H44A	−0.0557	0.6940	0.0007	0.062*	
H44B	−0.0539	0.5726	−0.0006	0.062*	
C34	0.2893 (3)	0.6290 (3)	0.05843 (12)	0.0545 (10)	
H34A	0.3405	0.5786	0.0453	0.065*	
H34B	0.3013	0.6931	0.0415	0.065*	
C28	0.3778 (3)	0.6752 (3)	0.20437 (15)	0.0655 (11)	
H28	0.3978	0.6851	0.2362	0.079*	
C33	0.1711 (3)	0.5936 (3)	0.04966 (12)	0.0512 (9)	
H33A	0.1534	0.6005	0.0159	0.061*	
H33B	0.1643	0.5217	0.0582	0.061*	
C43	−0.2025 (3)	0.6300 (3)	0.02647 (14)	0.0548 (9)	
C45	−0.2860 (3)	0.5952 (3)	−0.00045 (14)	0.0592 (10)	
H45	−0.3557	0.5948	0.0143	0.071*	
C35	0.3160 (3)	0.6445 (3)	0.11030 (12)	0.0453 (8)	
C41	−0.1050 (3)	0.8121 (3)	0.08368 (15)	0.0709 (12)	
H41A	−0.1602	0.8498	0.1014	0.106*	
H41B	−0.1186	0.8199	0.0500	0.106*	
H41C	−0.0320	0.8379	0.0913	0.106*	
C29	0.2674 (3)	0.6686 (3)	0.19177 (13)	0.0587 (10)	
H29	0.2135	0.6744	0.2156	0.070*	
C42	−0.2216 (3)	0.6622 (3)	0.07671 (14)	0.0575 (10)	
C39	−0.1118 (3)	0.6975 (3)	0.09718 (14)	0.0510 (10)	
C47	−0.2059 (4)	0.5875 (3)	−0.08312 (16)	0.0723 (12)	
H47	−0.1483	0.6313	−0.0740	0.087*	
C38	−0.0934 (3)	0.6821 (4)	0.14968 (14)	0.0695 (12)	
H38A	−0.1142	0.6125	0.1583	0.083*	
H38B	−0.1409	0.7289	0.1674	0.083*	
C48	−0.2113 (4)	0.5541 (4)	−0.12928 (18)	0.0925 (15)	
H48	−0.1593	0.5788	−0.1512	0.111*	
C46	−0.2847 (3)	0.5574 (3)	−0.04950 (15)	0.0604 (11)	
C49	−0.2898 (5)	0.4861 (4)	−0.14458 (18)	0.0908 (15)	
C51	−0.3674 (4)	0.4912 (4)	−0.06468 (19)	0.0855 (14)	
H51	−0.4234	0.4712	−0.0435	0.103*	
C50	−0.3677 (4)	0.4536 (4)	−0.11210 (19)	0.0962 (17)	
H50	−0.4216	0.4062	−0.1214	0.115*	

### Atomic displacement parameters (Å^2^)

**Table:**

	*U*^11^	*U*^22^	*U*^33^	*U*^12^	*U*^13^	*U*^23^
S2	0.266 (3)	0.177 (2)	0.0823 (12)	−0.066 (2)	−0.0282 (14)	0.0141 (12)
C52	0.152 (7)	0.215 (10)	0.224 (9)	0.006 (7)	0.053 (6)	0.111 (8)
S1	0.0891 (7)	0.0926 (9)	0.0500 (6)	0.0235 (7)	0.0109 (6)	0.0077 (6)
C10	0.0372 (17)	0.058 (3)	0.049 (2)	0.0036 (17)	−0.0041 (16)	0.0086 (18)
C1	0.0393 (18)	0.047 (2)	0.051 (2)	0.0058 (16)	0.0015 (16)	0.0093 (17)
C4	0.0404 (17)	0.042 (2)	0.043 (2)	−0.0010 (16)	0.0019 (15)	0.0042 (15)
O1	0.0374 (13)	0.084 (2)	0.0597 (17)	−0.0024 (13)	0.0078 (11)	0.0178 (15)
C11	0.0418 (17)	0.065 (3)	0.040 (2)	−0.0021 (18)	−0.0088 (15)	0.0044 (18)
C18	0.0374 (18)	0.078 (3)	0.041 (2)	−0.0095 (18)	0.0011 (15)	0.0042 (19)
C13	0.0359 (17)	0.047 (2)	0.043 (2)	−0.0053 (15)	−0.0040 (14)	0.0111 (17)
C2	0.0494 (19)	0.054 (2)	0.043 (2)	0.0037 (18)	0.0071 (16)	0.0108 (17)
C5	0.0411 (17)	0.046 (2)	0.040 (2)	−0.0003 (16)	−0.0070 (15)	0.0026 (15)
C3	0.0474 (19)	0.049 (2)	0.043 (2)	0.0007 (17)	−0.0042 (16)	−0.0012 (16)
C14	0.0363 (16)	0.045 (2)	0.040 (2)	−0.0087 (15)	−0.0017 (14)	0.0021 (15)
C16	0.0366 (18)	0.061 (3)	0.052 (2)	0.0019 (17)	−0.0032 (16)	0.0087 (18)
C8	0.0373 (17)	0.098 (3)	0.039 (2)	0.003 (2)	−0.0047 (15)	0.010 (2)
O2	0.0355 (13)	0.103 (2)	0.0654 (18)	−0.0082 (14)	−0.0083 (12)	0.0224 (15)
C22	0.075 (3)	0.059 (3)	0.059 (3)	−0.021 (2)	0.019 (2)	0.000 (2)
C20	0.0408 (19)	0.055 (2)	0.050 (2)	0.0003 (17)	0.0100 (16)	0.0024 (18)
C6	0.0360 (16)	0.048 (2)	0.039 (2)	−0.0029 (16)	−0.0007 (14)	0.0047 (16)
C12	0.0400 (18)	0.070 (3)	0.047 (2)	−0.0071 (18)	−0.0084 (15)	0.0100 (19)
C19	0.0401 (19)	0.066 (3)	0.050 (2)	−0.0013 (18)	−0.0021 (16)	0.0046 (18)
C24	0.052 (2)	0.103 (4)	0.049 (3)	−0.007 (2)	0.0033 (18)	−0.002 (2)
C17	0.0352 (17)	0.056 (2)	0.051 (2)	−0.0028 (16)	0.0036 (16)	0.0068 (18)
C9	0.0401 (18)	0.052 (2)	0.038 (2)	0.0042 (17)	−0.0036 (14)	−0.0014 (17)
C7	0.0399 (18)	0.079 (3)	0.047 (2)	0.0043 (19)	−0.0008 (15)	0.015 (2)
C23	0.059 (2)	0.062 (3)	0.046 (2)	0.015 (2)	0.011 (2)	−0.0047 (19)
C25	0.059 (2)	0.080 (3)	0.054 (3)	−0.020 (2)	0.0096 (19)	−0.004 (2)
C15	0.054 (2)	0.055 (3)	0.076 (3)	0.0048 (19)	0.0101 (19)	0.011 (2)
C21	0.057 (2)	0.081 (3)	0.052 (3)	−0.019 (2)	0.0031 (19)	0.001 (2)
C26	0.117 (4)	0.077 (3)	0.074 (3)	0.010 (3)	0.033 (3)	0.018 (2)
C36	0.046 (2)	0.049 (2)	0.060 (3)	0.0026 (17)	−0.0006 (17)	−0.0064 (19)
O3	0.0513 (15)	0.084 (2)	0.093 (2)	−0.0072 (16)	−0.0204 (14)	−0.0098 (19)
C32	0.0381 (16)	0.039 (2)	0.050 (2)	0.0018 (16)	0.0023 (15)	−0.0007 (16)
C40	0.0375 (17)	0.038 (2)	0.061 (2)	−0.0016 (15)	−0.0020 (16)	0.0011 (17)
C30	0.0427 (18)	0.042 (2)	0.048 (2)	−0.0037 (17)	0.0020 (16)	−0.0010 (17)
C31	0.0423 (18)	0.052 (2)	0.052 (2)	−0.0063 (17)	0.0039 (15)	−0.0015 (18)
C27	0.047 (2)	0.055 (3)	0.069 (3)	−0.0088 (19)	−0.012 (2)	−0.004 (2)
O4	0.0393 (14)	0.102 (2)	0.098 (2)	−0.0063 (16)	0.0093 (14)	−0.0180 (18)
C37	0.049 (2)	0.105 (4)	0.060 (3)	0.002 (2)	0.0116 (18)	−0.020 (2)
C44	0.0417 (18)	0.052 (2)	0.061 (2)	0.0005 (17)	−0.0034 (16)	−0.0047 (18)
C34	0.0405 (18)	0.068 (3)	0.055 (2)	0.0065 (18)	0.0024 (16)	−0.0060 (19)
C28	0.063 (3)	0.078 (3)	0.056 (3)	−0.013 (2)	−0.014 (2)	−0.009 (2)
C33	0.0415 (18)	0.060 (2)	0.052 (2)	0.0012 (18)	−0.0022 (16)	−0.0078 (17)
C43	0.0424 (19)	0.048 (2)	0.074 (3)	0.0036 (17)	−0.0044 (18)	0.0006 (19)
C45	0.041 (2)	0.061 (3)	0.076 (3)	−0.0011 (18)	−0.0092 (18)	0.005 (2)
C35	0.0393 (17)	0.041 (2)	0.055 (2)	−0.0013 (17)	−0.0026 (16)	0.0007 (16)
C41	0.056 (2)	0.048 (3)	0.109 (4)	0.009 (2)	0.002 (2)	−0.012 (2)
C29	0.058 (2)	0.066 (3)	0.052 (3)	−0.009 (2)	0.0023 (18)	−0.004 (2)
C42	0.0329 (19)	0.054 (2)	0.085 (3)	−0.0016 (17)	0.0021 (18)	−0.004 (2)
C39	0.0396 (18)	0.050 (3)	0.063 (3)	0.0009 (17)	0.0054 (16)	−0.0060 (18)
C47	0.066 (3)	0.070 (3)	0.081 (3)	0.005 (2)	−0.010 (2)	0.002 (2)
C38	0.043 (2)	0.097 (4)	0.068 (3)	−0.005 (2)	0.0153 (19)	−0.017 (2)
C48	0.094 (4)	0.105 (4)	0.078 (4)	0.000 (3)	−0.011 (3)	0.010 (3)
C46	0.049 (2)	0.054 (3)	0.078 (3)	0.007 (2)	−0.018 (2)	0.011 (2)
C49	0.105 (4)	0.090 (4)	0.078 (3)	−0.006 (3)	−0.018 (3)	0.013 (3)
C51	0.067 (3)	0.094 (4)	0.096 (4)	−0.018 (3)	−0.033 (3)	0.019 (3)
C50	0.109 (4)	0.090 (4)	0.089 (4)	−0.029 (3)	−0.056 (3)	0.009 (3)

### Geometric parameters (Å, °)

**Table:**

S2—C52	1.562 (8)	C15—H15C	0.9600
S2—C49	1.794 (6)	C21—H21	0.9300
C52—H52A	0.9600	C26—H26A	0.9600
C52—H52B	0.9600	C26—H26B	0.9600
C52—H52C	0.9600	C26—H26C	0.9600
S1—C26	1.754 (5)	C36—C27	1.362 (5)
S1—C23	1.756 (4)	C36—C35	1.393 (5)
C10—C1	1.369 (5)	C36—H36	0.9300
C10—C9	1.389 (4)	O3—C27	1.373 (4)
C10—H10	0.9300	O3—H3A	0.8200
C1—O1	1.366 (4)	C32—C33	1.501 (4)
C1—C2	1.384 (5)	C32—C40	1.518 (4)
C4—C3	1.388 (4)	C32—C31	1.521 (4)
C4—C9	1.395 (4)	C32—H32	0.9800
C4—C5	1.526 (4)	C40—C44	1.527 (5)
O1—H1	0.8200	C40—C39	1.530 (5)
C11—C5	1.532 (4)	C40—H40	0.9800
C11—C12	1.532 (4)	C30—C29	1.385 (5)
C11—H11A	0.9700	C30—C35	1.391 (4)
C11—H11B	0.9700	C30—C31	1.521 (4)
C18—C17	1.505 (4)	C31—C37	1.537 (5)
C18—C14	1.517 (4)	C31—H31	0.9800
C18—H18A	0.9700	C27—C28	1.371 (5)
C18—H18B	0.9700	O4—C42	1.218 (4)
C13—C12	1.506 (5)	C37—C38	1.541 (5)
C13—C16	1.513 (5)	C37—H37A	0.9700
C13—C15	1.530 (5)	C37—H37B	0.9700
C13—C14	1.542 (4)	C44—C43	1.511 (5)
C2—C3	1.383 (4)	C44—H44A	0.9700
C2—H2	0.9300	C44—H44B	0.9700
C5—C6	1.514 (4)	C34—C35	1.498 (5)
C5—H5	0.9800	C34—C33	1.508 (4)
C3—H3	0.9300	C34—H34A	0.9700
C14—C6	1.521 (4)	C34—H34B	0.9700
C14—H14	0.9800	C28—C29	1.370 (5)
C16—O2	1.227 (4)	C28—H28	0.9300
C16—C17	1.464 (5)	C33—H33A	0.9700
C8—C7	1.506 (5)	C33—H33B	0.9700
C8—C9	1.513 (4)	C43—C45	1.331 (5)
C8—H8A	0.9700	C43—C42	1.483 (5)
C8—H8B	0.9700	C45—C46	1.456 (5)
C22—C23	1.357 (5)	C45—H45	0.9300
C22—C21	1.390 (5)	C41—C39	1.542 (5)
C22—H22	0.9300	C41—H41A	0.9600
C20—C21	1.377 (5)	C41—H41B	0.9600
C20—C25	1.389 (5)	C41—H41C	0.9600
C20—C19	1.448 (5)	C29—H29	0.9300
C6—C7	1.521 (4)	C42—C39	1.505 (5)
C6—H6	0.9800	C39—C38	1.497 (5)
C12—H12A	0.9700	C47—C48	1.363 (6)
C12—H12B	0.9700	C47—C46	1.388 (5)
C19—C17	1.335 (4)	C47—H47	0.9300
C19—H19	0.9300	C38—H38A	0.9700
C24—C25	1.349 (5)	C38—H38B	0.9700
C24—C23	1.377 (5)	C48—C49	1.360 (7)
C24—H24	0.9300	C48—H48	0.9300
C7—H7A	0.9700	C46—C51	1.380 (6)
C7—H7B	0.9700	C49—C50	1.368 (7)
C25—H25	0.9300	C51—C50	1.413 (7)
C15—H15A	0.9600	C51—H51	0.9300
C15—H15B	0.9600	C50—H50	0.9300
			
C52—S2—C49	97.0 (4)	C22—C21—H21	119.3
S2—C52—H52A	109.5	S1—C26—H26A	109.5
S2—C52—H52B	109.5	S1—C26—H26B	109.5
H52A—C52—H52B	109.5	H26A—C26—H26B	109.5
S2—C52—H52C	109.5	S1—C26—H26C	109.5
H52A—C52—H52C	109.5	H26A—C26—H26C	109.5
H52B—C52—H52C	109.5	H26B—C26—H26C	109.5
C26—S1—C23	104.4 (2)	C27—C36—C35	121.6 (3)
C1—C10—C9	121.9 (3)	C27—C36—H36	119.2
C1—C10—H10	119.0	C35—C36—H36	119.2
C9—C10—H10	119.0	C27—O3—H3A	109.5
O1—C1—C10	123.3 (3)	C33—C32—C40	113.4 (3)
O1—C1—C2	117.6 (3)	C33—C32—C31	110.3 (3)
C10—C1—C2	119.1 (3)	C40—C32—C31	108.0 (3)
C3—C4—C9	117.7 (3)	C33—C32—H32	108.3
C3—C4—C5	121.5 (3)	C40—C32—H32	108.3
C9—C4—C5	120.8 (3)	C31—C32—H32	108.3
C1—O1—H1	109.5	C32—C40—C44	121.4 (3)
C5—C11—C12	112.3 (3)	C32—C40—C39	112.1 (3)
C5—C11—H11A	109.1	C44—C40—C39	104.5 (3)
C12—C11—H11A	109.1	C32—C40—H40	105.9
C5—C11—H11B	109.1	C44—C40—H40	105.9
C12—C11—H11B	109.1	C39—C40—H40	105.9
H11A—C11—H11B	107.9	C29—C30—C35	117.3 (3)
C17—C18—C14	102.3 (3)	C29—C30—C31	122.0 (3)
C17—C18—H18A	111.3	C35—C30—C31	120.5 (3)
C14—C18—H18A	111.3	C30—C31—C32	112.0 (3)
C17—C18—H18B	111.3	C30—C31—C37	113.9 (3)
C14—C18—H18B	111.3	C32—C31—C37	112.7 (3)
H18A—C18—H18B	109.2	C30—C31—H31	105.8
C12—C13—C16	118.3 (3)	C32—C31—H31	105.8
C12—C13—C15	112.0 (3)	C37—C31—H31	105.8
C16—C13—C15	104.8 (3)	C36—C27—C28	119.2 (3)
C12—C13—C14	108.3 (3)	C36—C27—O3	123.1 (4)
C16—C13—C14	99.8 (3)	C28—C27—O3	117.6 (3)
C15—C13—C14	113.2 (3)	C31—C37—C38	112.1 (3)
C3—C2—C1	119.3 (3)	C31—C37—H37A	109.2
C3—C2—H2	120.3	C38—C37—H37A	109.2
C1—C2—H2	120.3	C31—C37—H37B	109.2
C6—C5—C4	111.9 (3)	C38—C37—H37B	109.2
C6—C5—C11	111.7 (3)	H37A—C37—H37B	107.9
C4—C5—C11	114.2 (3)	C43—C44—C40	101.8 (3)
C6—C5—H5	106.1	C43—C44—H44A	111.4
C4—C5—H5	106.1	C40—C44—H44A	111.4
C11—C5—H5	106.1	C43—C44—H44B	111.4
C2—C3—C4	122.3 (3)	C40—C44—H44B	111.4
C2—C3—H3	118.8	H44A—C44—H44B	109.3
C4—C3—H3	118.8	C35—C34—C33	113.5 (3)
C18—C14—C6	122.4 (3)	C35—C34—H34A	108.9
C18—C14—C13	105.2 (3)	C33—C34—H34A	108.9
C6—C14—C13	111.5 (3)	C35—C34—H34B	108.9
C18—C14—H14	105.5	C33—C34—H34B	108.9
C6—C14—H14	105.5	H34A—C34—H34B	107.7
C13—C14—H14	105.5	C29—C28—C27	119.8 (4)
O2—C16—C17	125.8 (3)	C29—C28—H28	120.1
O2—C16—C13	125.3 (3)	C27—C28—H28	120.1
C17—C16—C13	108.9 (3)	C32—C33—C34	110.7 (3)
C7—C8—C9	113.5 (3)	C32—C33—H33A	109.5
C7—C8—H8A	108.9	C34—C33—H33A	109.5
C9—C8—H8A	108.9	C32—C33—H33B	109.5
C7—C8—H8B	108.9	C34—C33—H33B	109.5
C9—C8—H8B	108.9	H33A—C33—H33B	108.1
H8A—C8—H8B	107.7	C45—C43—C42	121.0 (3)
C23—C22—C21	120.8 (4)	C45—C43—C44	130.7 (4)
C23—C22—H22	119.6	C42—C43—C44	107.7 (3)
C21—C22—H22	119.6	C43—C45—C46	129.8 (4)
C21—C20—C25	116.4 (3)	C43—C45—H45	115.1
C21—C20—C19	120.4 (3)	C46—C45—H45	115.1
C25—C20—C19	123.1 (3)	C30—C35—C36	119.6 (3)
C5—C6—C7	110.6 (3)	C30—C35—C34	122.2 (3)
C5—C6—C14	108.0 (3)	C36—C35—C34	118.1 (3)
C7—C6—C14	113.0 (3)	C39—C41—H41A	109.5
C5—C6—H6	108.4	C39—C41—H41B	109.5
C7—C6—H6	108.4	H41A—C41—H41B	109.5
C14—C6—H6	108.4	C39—C41—H41C	109.5
C13—C12—C11	111.1 (3)	H41A—C41—H41C	109.5
C13—C12—H12A	109.4	H41B—C41—H41C	109.5
C11—C12—H12A	109.4	C28—C29—C30	122.5 (4)
C13—C12—H12B	109.4	C28—C29—H29	118.7
C11—C12—H12B	109.4	C30—C29—H29	118.7
H12A—C12—H12B	108.0	O4—C42—C43	125.5 (3)
C17—C19—C20	128.4 (3)	O4—C42—C39	126.4 (4)
C17—C19—H19	115.8	C43—C42—C39	108.1 (3)
C20—C19—H19	115.8	C38—C39—C42	117.4 (3)
C25—C24—C23	121.9 (4)	C38—C39—C40	110.3 (3)
C25—C24—H24	119.1	C42—C39—C40	100.0 (3)
C23—C24—H24	119.1	C38—C39—C41	111.2 (3)
C19—C17—C16	122.1 (3)	C42—C39—C41	104.4 (3)
C19—C17—C18	129.4 (3)	C40—C39—C41	113.2 (3)
C16—C17—C18	108.3 (3)	C48—C47—C46	121.2 (5)
C10—C9—C4	119.7 (3)	C48—C47—H47	119.4
C10—C9—C8	118.3 (3)	C46—C47—H47	119.4
C4—C9—C8	122.0 (3)	C39—C38—C37	111.3 (3)
C8—C7—C6	110.0 (3)	C39—C38—H38A	109.4
C8—C7—H7A	109.7	C37—C38—H38A	109.4
C6—C7—H7A	109.7	C39—C38—H38B	109.4
C8—C7—H7B	109.7	C37—C38—H38B	109.4
C6—C7—H7B	109.7	H38A—C38—H38B	108.0
H7A—C7—H7B	108.2	C49—C48—C47	122.6 (5)
C22—C23—C24	117.8 (4)	C49—C48—H48	118.7
C22—C23—S1	125.6 (3)	C47—C48—H48	118.7
C24—C23—S1	116.6 (3)	C51—C46—C47	117.1 (4)
C24—C25—C20	121.6 (4)	C51—C46—C45	119.5 (4)
C24—C25—H25	119.2	C47—C46—C45	123.3 (4)
C20—C25—H25	119.2	C48—C49—C50	117.6 (5)
C13—C15—H15A	109.5	C48—C49—S2	116.3 (4)
C13—C15—H15B	109.5	C50—C49—S2	126.0 (4)
H15A—C15—H15B	109.5	C46—C51—C50	120.4 (5)
C13—C15—H15C	109.5	C46—C51—H51	119.8
H15A—C15—H15C	109.5	C50—C51—H51	119.8
H15B—C15—H15C	109.5	C49—C50—C51	120.9 (5)
C20—C21—C22	121.5 (4)	C49—C50—H50	119.6
C20—C21—H21	119.3	C51—C50—H50	119.6
			
C9—C10—C1—O1	179.9 (3)	C33—C32—C40—C44	−53.6 (4)
C9—C10—C1—C2	−0.9 (6)	C31—C32—C40—C44	−176.1 (3)
O1—C1—C2—C3	179.0 (3)	C33—C32—C40—C39	−178.0 (3)
C10—C1—C2—C3	−0.2 (5)	C31—C32—C40—C39	59.5 (4)
C3—C4—C5—C6	162.7 (3)	C29—C30—C31—C32	163.3 (3)
C9—C4—C5—C6	−19.3 (5)	C35—C30—C31—C32	−21.8 (5)
C3—C4—C5—C11	34.4 (5)	C29—C30—C31—C37	34.0 (5)
C9—C4—C5—C11	−147.6 (3)	C35—C30—C31—C37	−151.1 (4)
C12—C11—C5—C6	53.5 (4)	C33—C32—C31—C30	50.3 (4)
C12—C11—C5—C4	−178.1 (3)	C40—C32—C31—C30	174.8 (3)
C1—C2—C3—C4	0.8 (6)	C33—C32—C31—C37	−179.7 (3)
C9—C4—C3—C2	−0.3 (5)	C40—C32—C31—C37	−55.3 (4)
C5—C4—C3—C2	177.8 (3)	C35—C36—C27—C28	−1.5 (6)
C17—C18—C14—C6	−164.5 (3)	C35—C36—C27—O3	176.1 (4)
C17—C18—C14—C13	−36.0 (4)	C30—C31—C37—C38	−178.6 (3)
C12—C13—C14—C18	163.1 (3)	C32—C31—C37—C38	52.4 (5)
C16—C13—C14—C18	38.8 (3)	C32—C40—C44—C43	−165.7 (3)
C15—C13—C14—C18	−72.0 (3)	C39—C40—C44—C43	−37.9 (3)
C12—C13—C14—C6	−62.2 (4)	C36—C27—C28—C29	0.4 (7)
C16—C13—C14—C6	173.5 (3)	O3—C27—C28—C29	−177.4 (4)
C15—C13—C14—C6	62.7 (4)	C40—C32—C33—C34	175.1 (3)
C12—C13—C16—O2	38.0 (6)	C31—C32—C33—C34	−63.6 (4)
C15—C13—C16—O2	−87.6 (5)	C35—C34—C33—C32	45.8 (4)
C14—C13—C16—O2	155.0 (4)	C40—C44—C43—C45	−153.0 (4)
C12—C13—C16—C17	−144.2 (3)	C40—C44—C43—C42	18.8 (4)
C15—C13—C16—C17	90.1 (3)	C42—C43—C45—C46	−178.1 (4)
C14—C13—C16—C17	−27.2 (4)	C44—C43—C45—C46	−7.3 (7)
C4—C5—C6—C7	50.0 (4)	C29—C30—C35—C36	−1.7 (5)
C11—C5—C6—C7	179.6 (3)	C31—C30—C35—C36	−177.0 (3)
C4—C5—C6—C14	174.1 (3)	C29—C30—C35—C34	−179.6 (3)
C11—C5—C6—C14	−56.3 (4)	C31—C30—C35—C34	5.2 (5)
C18—C14—C6—C5	−172.8 (3)	C27—C36—C35—C30	2.3 (6)
C13—C14—C6—C5	61.5 (4)	C27—C36—C35—C34	−179.8 (3)
C18—C14—C6—C7	−50.1 (5)	C33—C34—C35—C30	−17.2 (5)
C13—C14—C6—C7	−175.8 (3)	C33—C34—C35—C36	165.0 (3)
C16—C13—C12—C11	168.9 (3)	C27—C28—C29—C30	0.1 (7)
C15—C13—C12—C11	−69.1 (4)	C35—C30—C29—C28	0.6 (6)
C14—C13—C12—C11	56.5 (4)	C31—C30—C29—C28	175.8 (4)
C5—C11—C12—C13	−53.6 (4)	C45—C43—C42—O4	0.1 (6)
C21—C20—C19—C17	158.8 (4)	C44—C43—C42—O4	−172.6 (4)
C25—C20—C19—C17	−21.5 (6)	C45—C43—C42—C39	180.0 (4)
C20—C19—C17—C16	−177.9 (4)	C44—C43—C42—C39	7.3 (4)
C20—C19—C17—C18	−3.9 (7)	O4—C42—C39—C38	30.7 (6)
O2—C16—C17—C19	−1.2 (6)	C43—C42—C39—C38	−149.1 (3)
C13—C16—C17—C19	−179.0 (4)	O4—C42—C39—C40	149.9 (4)
O2—C16—C17—C18	−176.3 (4)	C43—C42—C39—C40	−29.9 (4)
C13—C16—C17—C18	5.9 (4)	O4—C42—C39—C41	−92.9 (5)
C14—C18—C17—C19	−156.0 (4)	C43—C42—C39—C41	87.3 (3)
C14—C18—C17—C16	18.6 (4)	C32—C40—C39—C38	−60.6 (4)
C1—C10—C9—C4	1.5 (6)	C44—C40—C39—C38	166.1 (3)
C1—C10—C9—C8	−179.1 (4)	C32—C40—C39—C42	175.1 (3)
C3—C4—C9—C10	−0.8 (5)	C44—C40—C39—C42	41.8 (4)
C5—C4—C9—C10	−178.9 (3)	C32—C40—C39—C41	64.6 (4)
C3—C4—C9—C8	179.7 (3)	C44—C40—C39—C41	−68.7 (4)
C5—C4—C9—C8	1.6 (5)	C42—C39—C38—C37	168.4 (4)
C7—C8—C9—C10	165.4 (3)	C40—C39—C38—C37	54.8 (4)
C7—C8—C9—C4	−15.2 (5)	C41—C39—C38—C37	−71.5 (4)
C9—C8—C7—C6	45.5 (5)	C31—C37—C38—C39	−51.5 (5)
C5—C6—C7—C8	−64.4 (4)	C46—C47—C48—C49	−3.4 (7)
C14—C6—C7—C8	174.4 (3)	C48—C47—C46—C51	1.0 (6)
C21—C22—C23—C24	−0.4 (6)	C48—C47—C46—C45	−176.7 (4)
C21—C22—C23—S1	179.1 (3)	C43—C45—C46—C51	155.9 (4)
C25—C24—C23—C22	1.5 (6)	C43—C45—C46—C47	−26.5 (6)
C25—C24—C23—S1	−178.0 (3)	C47—C48—C49—C50	2.2 (8)
C26—S1—C23—C22	18.8 (4)	C47—C48—C49—S2	−179.6 (4)
C26—S1—C23—C24	−161.7 (3)	C52—S2—C49—C48	167.6 (5)
C23—C24—C25—C20	−0.3 (7)	C52—S2—C49—C50	−14.4 (6)
C21—C20—C25—C24	−2.0 (6)	C47—C46—C51—C50	2.3 (6)
C19—C20—C25—C24	178.3 (4)	C45—C46—C51—C50	−179.9 (4)
C25—C20—C21—C22	3.0 (6)	C48—C49—C50—C51	1.2 (8)
C19—C20—C21—C22	−177.3 (4)	S2—C49—C50—C51	−176.8 (4)
C23—C22—C21—C20	−1.9 (6)	C46—C51—C50—C49	−3.5 (7)

### Hydrogen-bond geometry (Å, °)

**Table:**

*D*—H···*A*	*D*—H	H···*A*	*D*···*A*	*D*—H···*A*
C19—H19···O2	0.93	2.57	2.894 (4)	101
C45—H45···O4	0.93	2.53	2.880 (5)	103
O1—H1···O2^i^	0.82	1.95	2.746 (3)	163
C10—H10···O2^i^	0.93	2.53	3.179 (4)	127
O3—H3A···O4^ii^	0.82	2.03	2.830 (4)	166
C36—H36···O4^ii^	0.93	2.47	3.209 (4)	136

Symmetry codes: (i) *x*−1, *y*, *z*; (ii) *x*+1, *y*, *z*.

## References

[bb1] Alvarez-Ginarte, Y. M., Crespo, R., Montero-Cabrera, L. A., Ruiz-Garcia, J. A., Ponce, Y. M., Santana, R., Pardillo-Fontdevila, E. & Alonso-Becerra, E. (2005). *QSAR Comb. Sci.* **24**, 218–226.

[bb2] Bruker (2004). *APEX2* Bruker AXS Inc., Madison, Wisconsin, USA.

[bb3] Cremer, D. & Pople, J. A. (1975). *J. Am. Chem. Soc.* **97**, 1354–1358.

[bb4] Flack, H. D. (1983). *Acta Cryst.* A**39**, 876–881.

[bb5] Sheldrick, G. M. (1996). *SADABS*, University of Göttingen, Germany.

[bb6] Sheldrick, G. M. (2008). *Acta Cryst.* A**64**, 112–122. 10.1107/S0108767307043930 18156677

[bb7] Spek, A. L. (2003). *J. Appl. Cryst.* **36**, 7–13.

[bb8] Suitchmezian, V., Jess, I. & Näther, C. (2007). *Acta Cryst.* E**63**, o4839.

[bb9] Ye, Y.-Y. (2007). *Acta Cryst.* E**63**, o3022.

